# Propensity score-matched comparison of safety outcomes between high-risk and low-risk patients towards early hospital discharge after laparoscopic cholecystectomy

**DOI:** 10.1097/MS9.0000000000001300

**Published:** 2023-09-13

**Authors:** Siripong Cheewatanakornkul, Kamthorn Yolsuriyanwong, Piyanun Wangkulangkul, Praisuda Bualoy, Kanittha Sakolprakaikit

**Affiliations:** a Department of Surgery, Songklanagarind Hospital, Faculty of Medicine; b Department of Surgical Nursing, Faculty of Nursing, Prince of Songkla University, Songkhla, Thailand

**Keywords:** early hospital discharge, high-risk patients, laparoscopic cholecystectomy, propensity score matching, safety outcomes

## Abstract

**Background:**

Laparoscopic cholecystectomy (LC), a common treatment for symptomatic gallstones, has demonstrated safety in low-risk patients. However, existing data are scarce regarding the safety of LC in high-risk patients and the feasibility of early hospital discharge.

**Materials and methods:**

This retrospective study included 2296 patients diagnosed with symptomatic gallstones who underwent LC at a tertiary care centre from January 2009 through December 2019. The authors employed propensity score matching to mitigate bias between groups. Statistical significance was set at *P* less than 0.05.

**Results:**

The median age of the patients was 56 years (range 46–67), with a mean BMI of 25.2±4.3 kg/m^2^. Patients were classified as: American Society of Anesthesiologists (ASA) I (19.7%), II (68.3%), III (12.0%), and IV (0%). ASA I–II included low surgical risk patients (88%) and ASA III–IV comprised high-risk patients (12%). The LC-related 30-day reoperative rate was 0.2% and the readmission rate was 0.87%. Nine patients (0.4%) sustained major bile duct injuries, resulting in a conversion rate of 2.4%. The postoperative mortality rate was 0.04%, and the mean hospitalization time was 3.5 days. Patients in the high-risk group with a history of acute cholecystitis exhibited greater estimated blood loss, longer operative times, and were significantly more likely to be in the longer-stay group.

**Conclusion:**

These findings suggest that LC can be conducted safely on high-risk patients, and early hospital discharge is achievable. However, specific factors, such as a history of acute cholecystitis, may result in prolonged hospitalization owing to increased blood loss and longer operative times.

## Introduction

Highlights

- This retrospective study included 2296 patients with symptomatic gallstones who underwent laparoscopic cholecystectomy (LC) at a tertiary care centre between 2009 and 2019.
- LC was found to be safe in both low- and high-risk patients, with low 30-day reoperation (0.2%) and readmission rates (0.87%).
- Early hospital discharge is feasible for high-risk patients undergoing LC and cost-effective.
- Factors such as a history of acute cholecystitis may result in longer hospitalizations.

Gallstone disease affects 10–20% of adults^1^. with laparoscopic cholecystectomy (LC) being the standard treatment^2,3^. LC offers advantages such as improved cosmetic outcomes and faster recovery^4^. It is considered safe for high-risk patients^5,6^.

Ambulatory LC has been proven safe and cost-effective since the early 1990s^7,8^. but its acceptance, especially in developing countries, has been limited due to concerns about postoperative complications^9,10^. Therefore, establishing principles for optimizing ambulatory procedures is essential for success and patient safety. Although the safety and feasibility of ambulatory LC have been reported for low-risk and high-risk patients^11–15^, there is a lack of studies specifically focusing on high-risk patients. Ambulatory LC remains uncommon for patients classified as American Society of Anesthesiologists (ASA) grade III.

In Thailand, since 2018, public hospitals have implemented 1-day surgery programs, including ambulatory LC, supported by the government^16^. This policy aims to enhance patient recovery, satisfaction, and cost-effectiveness by allowing suitable patients to be discharged within 6 h^17,18^. However, careful patient selection is crucial for ambulatory LC, and its feasibility in high-risk patients has been inadequately explored^14,15^. Our study aims to report the experience of LC in a tertiary hospital, compare postoperative outcomes between high-risk and low-risk patients, and evaluate safety outcomes in early vs. late discharge groups among high-risk patients.

## Methods

### Research question and aims

The main focus of our study was threefold: Firstly, to provide a comprehensive overview of the LC experience within a tertiary hospital setting. Secondly, to conduct a propensity score comparison of postoperative outcomes between high-risk and low-risk patients who underwent LC. And finally, to assess the safety outcomes associated with early and late discharge within the high-risk patient group.

#### Data collection

Retrospective data were collected from gallstone-related symptom patients who underwent LC at a tertiary care hospital between January 2009 and December 2019. Exclusions included patients undergoing concurrent procedures, double gallbladder anatomy, or diagnosed with gallbladder cancer. Patient characteristics, laboratory results, preoperative ultrasonography findings, and surgical techniques were reviewed. Intraoperative and postoperative complications were classified using the Clavien-Dindo system. High-risk patients were defined as ASA greater than 2, while low-risk patients were ASA less than or equal to 2. Early discharge was defined as within 24 h postoperatively, and late discharge as after 24 h. LC procedures mainly used the conventional or alternative fundus-down technique based on surgeon judgment. Standard four-port approach with a 30° laparoscope was employed. Pneumoperitoneum was achieved using an open technique, maintaining intra-abdominal pressure at 12–15 mmHg. Postoperatively, patients were given a liquid or soft diet and discharged within one to 2 days. Follow-up appointments were recommended within 2 weeks or sooner for complications such as infection, abdominal pain, jaundice, or fever. Port-site hernia detection occurred during routine follow-up.

### Ethical approval

This study was approved by our Research Ethics Committee. The study design, data collection methods, and informed consent procedures were reviewed and approved by the committee. The study was conducted in accordance with the principles outlined in the Declaration of Helsinki and adhered to relevant national and institutional guidelines. Given the retrospective nature of our study, the need for informed consent was waived.

### Statistical analysis

Statistical analyses were conducted using R software version 4.1.1^19^. The Wilcoxon rank-sum test evaluated patient characteristic differences, while Pearson’s χ^2^ test compared categorical data. Normally distributed continuous variables had mean ± standard deviation calculated, while non-normally distributed variables had median and interquartile range reported. Propensity score matching (1:1 nearest neighbour matching) balanced patient characteristics and reduced survival analysis bias, accounting for age, sex, BMI, comorbidities (diabetes mellitus, hypertension, dyslipidemia, cerebrovascular disease, chronic obstructive pulmonary disease, cardiovascular disease, liver cirrhosis, and chronic kidney disease), history of acute cholecystitis, gallstone pancreatitis, cholangitis, endoscopic retrograde cholangiopancreatography (ERCP), leukocytosis, and imaging and intraoperative findings (acute cholecystitis, positive sonographic Murphy sign, liver cirrhosis, and gallbladder perforation). Results are reported in the table as unmatched or matched. A *P* value less than 0.05 indicated statistical significance.

## Results

We reviewed 2297 patients, including 1527 females (66.5%). The median age of the patients was 56 years (range: 46–67 years). The mean BMI was 25.2±4.3 kg/m^2^. The percentage of patients classified as ASA I, II, III, and IV was 19.7%, 68.3%, 12.0%, and 0%, respectively. The most common surgical indication was symptomatic cholelithiasis in 1724 cases (75.1%). Chronic and acute cholecystitis was observed in 212 (9.2%) and 167 (7.3%) patients, respectively. Common bile duct (CBD) stones and pancreatitis were observed in 91 (3.9%) and 79 (3.4%) patients, respectively. A history of multiple episodes (≥2 times) of biliary colic was found in 2214 patients (96.4%). Post-ERCP common bile duct stone clearance was observed in 500 (21.8%) patients. A history of acute cholecystitis was found in 365 (15.9%) patients, cholangitis in 183 (8%), and gallstone pancreatitis in 168 (7.3%). A history of upper abdominal surgery was found in 38 patients (1.7%). Ultrasonography, computed tomography, and magnetic resonance cholangiopancreatography were performed preoperatively in 2216 patients (96.5%), 390 (17%), and 140 (6.1%), respectively.

The infundibular approach was performed in 1624 patients (70.7%) and required the fundus-down technique in 673 patients (29.3%). The mean operative time was 131.7±47.6 minutes, and the mean hospital stay was 2.4±2.7 days. Major and minor bile duct injuries occurred in nine (0.4%) and 22 (0.9%) patients, respectively. Conversion from laparoscopy to open cholecystectomy was performed in 54 patients (2.4%). The causes for conversion were due to severe adhesions, bleeding, bile duct injury, and uncertain cystic duct stump closure in 44, 5, 3, and 2 patients, respectively. Postoperative mortality occurred in one patient (0.04%). Within 30 days, four patients (0.2%) required reoperation, and the LC-related 30-day readmission rate was 0.87%. The median follow-up time in this study was 24 days (range 14–131). During the follow-up period, 55 patients (2.4%) developed surgical site infections (which) treated with antibiotics. Trocar site hernia (TSH) was detected at the umbilical port site in 13 patients (0.6%). Other complications according to the Clavien-Dindo classification are shown in Supplement 1, Supplemental Digital Content 1, http://links.lww.com/MS9/A251.

Table 1 shows the comparison of patient characteristics according to the ASA classification between low-risk (ASA ≤2) and high-risk (ASA >2) patients categorized by unmatched and matched data. The operative outcomes of the 276 matched patients in each group were compared. Only the higher estimated blood loss in the high-risk group was statistically significant (10 vs. 5 ml; *P*=0.005). The length of hospital stay between the two groups was slightly different but not statistically significant (Table 2).

**Table 1:** Comparison of patient characteristics by ASA classification between low-risk (ASA ≤2) and high-risk (ASA >2) patients categorized as unmatched and matched data^a^

	Unmatched data			Matched data		
Characteristics	Low-risk (*n*=2020)	High-risk (*n*=276)	*P*	Low-risk (*n*= 76)	High-risk (*n*=276)	*P*
Demographic and clinical variables						
Age, median (range)	55 (45–65)	68.5 (58–76)	<0.001	69 (58–75)	68.5 (58–76)	0.46
Sex			<0.001			0.547
Female	647 (32)	123 (44.6)		161 (58.3)	153 (55.4)	
Male	1373 (68)	153 (55.4)		115 (41.7)	123 (44.6)	
BMI			0.001			0.084
<18.5	64 (3.2)	14 (5.1)		10 (3.6)	14 (5.1)	
18.5–22.9	580 (28.7)	62 (22.5)		76 (27.5)	62 (22.5)	
23–24.9	413 (20.4)	49 (17.8)		52 (18.8)	49 (17.8)	
25–29.9	724 (35.8)	97 (35.1)		106 (38.4)	97 (35.1)	
≥30	239 (11.8)	54 (19.6)		32 (11.6)	54 (19.6)	
Comorbidities	1010 (50)	248 (89.9)	<0.001	237 (85.9)	248 (89.9)	0.192
Diabetes mellitus	298 (14.8)	87 (31.5)	<0.001	95 (34.4)	87 (31.5)	0.526
Hypertension	564 (27.9)	159 (57.6)	<0.001	167 (60.5)	159 (57.6)	0.545
Dyslipidemia	398 (19.7)	109 (39.5)	<0.001	113 (40.9)	109 (39.5)	0.795
Cerebrovascular disease	29 (1.4)	16 (5.8)	<0.001	14 (5.1)	16 (5.8)	0.851
Chronic obstructive pulmonary disease	17 (0.8)	6 (2.2)	0.078	7 (2.5)	6 (2.2)	1
Cardiovascular disease	70 (3.5)	54 (19.6)	<0.001	51 (18.5)	54 (19.6)	0.828
Liver cirrhosis	32 (1.6)	18 (6.5)	<0.001	17 (6.2)	18 (6.5)	1
Chronic kidney disease	24 (1.2)	27 (9.8)	<0.001	17 (6.2)	27 (9.8)	0.157
History						
Acute cholecystitis	291 (14.4)	73 (26.4)	<0.001	77 (27.9)	73 (26.4)	0.774
Gallstone pancreatitis	139 (6.9)	29 (10.5)	0.041	25 (9.1)	29 (10.5)	0.667
Cholangitis	143 (7.1)	40 (14.5)	<0.001	46 (16.7)	40 (14.5)	0.557
ERCP	411 (20.3)	89 (32.2)	<0.001	80 (29)	89 (32.2)	0.46
≥2 episodes of biliary colic	1945 (96.3)	268 (97.1)	0.612	264 (95.7)	268 (97.1)	0.494
Upper abdominal surgery	31 (1.5)	7 (2.5)	0.331	7 (2.5)	7 (2.5)	1
Fever at an admission date (BT >38°C)	23 (1.1)	7 (2.5)	0.102	9 (3.3)	7 (2.5)	0.8
Laboratory						
Total bilirubin >1.5	126 (6.4)	25 (9.2)	0.116	23 (8.4)	25 (9.2)	0.859
SGOT >100	55 (2.8)	7 (2.6)	0.986	10 (3.6)	7 (2.6)	0.633
SGPT >100	83 (4.2)	4 (1.5)	0.042	13 (4.7)	4 (1.5)	0.05
ALP >150	118 (6)	25 (9.2)	0.061	26 (9.5)	25 (9.2)	1
WBC count >18 000	24 (1.2)	12 (4.3)	<0.001	13 (4.7)	12 (4.3)	1
Imaging and intraoperative findings						
Thickened gallbladder wall	383 (19)	62 (22.5)	0.194	72 (26.1)	62 (22.5)	0.372
Small contracted/shrunken/collapsed gallbladder	109 (5.4)	19 (6.9)	0.384	15 (5.4)	19 (6.9)	0.595
Distended gallbladder	165 (8.2)	34 (12.3)	0.029	40 (14.5)	34 (12.3)	0.532
Impacted stone at neck	60 (3)	11 (4)	0.466	14 (5.1)	11 (4)	0.682
Gangrenous gallbladder	10 (0.5)	4 (1.4)	0.134	4 (1.4)	4 (1.4)	1
Gallbladder perforation	16 (0.8)	8 (2.9)	0.004	6 (2.2)	8 (2.9)	0.787
Sonographic Murphy sign positive	91 (4.5)	26 (9.4)	<0.001	28 (10.1)	26 (9.4)	0.886
Acute cholecystitis	227 (11.2)	55 (19.9)	<0.001	55 (19.9)	55 (19.9)	1
Chronic cholecystitis	165 (8.2)	23 (8.3)	1	20 (7.2)	23 (8.3)	0.751
IHD of CBD dilation	254 (12.6)	45 (16.3)	0.103	49 (17.8)	45 (16.3)	0.734
Mirizzi syndrome/ cholecystoenteric fistula	11 (0.5)	0	0.445	1 (0.4)	0	1
Pancreatitis	35 (1.7)	6 (2.2)	0.782	6 (2.2)	6 (2.2)	1
Liver cirrhosis	33 (1.6)	14 (5.1)	<0.001	12 (4.3)	14 (5.1)	0.841
Cystic duct stone	53 (2.6)	9 (3.3)	0.678	7 (2.5)	9 (3.3)	0.8

a Matched data: age, sex, BMI, comorbidities (diabetes mellitus, hypertension, dyslipidemia, cerebrovascular disease, chronic obstructive pulmonary disease, cardiovascular disease, liver cirrhosis, and chronic kidney disease), history of (i) acute cholecystitis, (ii) gallstone pancreatitis, (iii) cholangitis, or (iv) ERCP, leukocytosis, acute cholecystitis from imaging and intraoperative findings, sonographically positive Murphy sign, liver cirrhosis, and gallbladder perforation.

Data are presented as *n* (%) unless otherwise specified.

ALP, alkaline phosphatase; ASA, American Society of Anesthesiologists; BT, body temperature; CBD, common bile duct; ERCP, endoscopic retrograde cholangiopancreatography; IHD, intrahepatic duct; SGOT, serum glutamate oxaloacetate transaminase; SGPT, serum glutamate pyruvate transaminase; WBC, white blood cell.

**Table 2:** Comparison of operative and postoperative outcomes in the low-risk and high-risk groups in a propensity score-matched patient population

	Low-risk (*n*=276)^a^	High-risk (*n*=276)^a^	*P*
Complication^b^ (Clavien-Dindo classification)	39 (14.1)	46 (16.7)	0.479
Grade I			
Surgical site infection	11 (4)	11 (4)	1
Wound dehiscence	2 (0.7)	2 (0.7)	1
Grade II			
Biliary pancreatitis	2 (0.7)	0	0.499
Grade IIIa–b			
Postoperative CBD stone	12 (4.3)	7 (2.5)	0.35
Liver injury grade I	6 (2.2)	5 (1.8)	1
Bile duct injury			
Major duct injury			
Common bile duct injury^c^	1 (0.4)	0	1
Hepatic duct injury	0	1 (0.4)	1
Minor duct injury			
Cystic duct injury	1 (0.4)	2 (0.7)	1
Cystic duct stump leakage	1 (0.4)	4 (1.4)	0.373
Duct of Luschka injury	2 (0.7)	0	0.499
Postoperative bile leakage	0	1 (0.4)	1
Bowel injury	1 (0.4)	4 (1.4)	0.373
Postoperative intra-abdominal collection	1 (0.4)	7 (2.5)	0.068
Port-site hernia	2 (0.7)	3 (1.1)	1
Cystic artery injury	1 (0.4)	3 (1.1)	0.624
Hepatic vessel injury	0	1 (0.4)	1
Estimated blood loss, mL, median (range)	5 (5–20)	10 (5–20)	0.005
Operative time, min, median (range)	130 (108.8–160)	135 (110–165)	0.372
Conversion	13 (4.7)	6 (2.2)	0.161
Length of stay, days, median (range)	3 (2–4)	3 (2–5)	0.056
30-day reoperation^c^	0	1 (0.4)	1
30-day readmission			
All-cause readmission	5 (1.8)	6 (2.2)	1
LC related	3 (1.1)	4 (1.4)	1

a Low and high-risk groups: ASA ≤2 and ASA >2.

b No complication in Clavien-Dindo classification grade V.

c A number of cases were not successfully matched during the propensity score matching process, leading to their exclusion from the subsequent analysis.

Data are presented as *n* (%) unless otherwise indicated.

CBD, common bile duct; LC, laparoscopic cholecystectomy.

Table 3 shows patient characteristics and operative results in the high-risk patient group divided into early and late hospital discharge. Patient characteristics including age, sex, BMI, and comorbidities between the two groups were not statistically different. However, patients with a history of acute cholecystitis detected from imaging or intraoperative findings, or multiple episodes of biliary colic required a longer hospital stays. Furthermore, patients experiencing a greater volume of estimated blood loss and a longer operative time were significantly more likely to be in the longer-stay group.

**Table 3:** Patient characteristics and operative results of high-risk patients with early and late hospital discharge^a^.

	Early discharge (*n*=69)	Late discharge (*n*=207)	*P*
Demographic and clinical variables			
Age, year (range)	68 (58–74)	69 (58.5–76)	0.602
Sex			0.834
Female	37 (53.6)	116 (56)	
Male	32 (46.4)	91 (44)	
BMI			0.455
<18.5	2 (2.9)	12 (5.8)	
18.5–22.9	20 (29)	42 (20.3)	
23–24.9	10 (14.5)	39 (18.8)	
25–29.9	22 (31.9)	75 (36.2)	
≥30	15 (21.7)	39 (18.8)	
Comorbidities			
Diabetes mellitus	19 (27.5)	68 (32.9)	0.501
Hypertension	38 (55.1)	121 (58.5)	0.725
Dyslipidemia	34 (49.3)	75 (36.2)	0.076
Cerebrovascular disease	2 (2.9)	14 (6.8)	0.372
Chronic obstructive pulmonary disease	0	6 (2.9)	0.342
Cardiovascular disease	14 (20.3)	40 (19.3)	1
Liver cirrhosis	5 (7.2)	13 (6.3)	0.781
Chronic kidney disease	8 (11.6)	19 (9.2)	0.726
History			
Acute cholecystitis	8 (11.6)	65 (31.4)	0.002
Gallstone pancreatitis	7 (10.1)	22 (10.6)	1
Cholangitis	8 (11.6)	32 (15.5)	0.554
ERCP	19 (27.5)	70 (33.8)	0.413
≥2 episodes of biliary colic	64 (92.8)	204 (98.6)	0.025
Upper abdominal surgery	3 (4.3)	4 (1.9)	0.372
Fever at an admission date (BT >38°C)	0	7 (3.4)	0.198
Laboratory results			
Total bilirubin > 1.5	3 (4.5)	22 (10.7)	0.195
SGOT >100	2 (3)	5 (2.4)	0.683
SGPT >100	1 (1.5)	3 (1.5)	1
ALP >150	7 (10.4)	18 (8.8)	0.868
WBC count >18 000	1 (1.4)	11 (5.3)	0.305
Imaging or intraoperative findings			
Thickened gallbladder wall	11 (15.9)	51 (24.6)	0.183
Small contracted/shrunken/collapsed gallbladder	2 (2.9)	17 (8.2)	0.173
Distended gallbladder	5 (7.2)	29 (14)	0.204
Impacted stone at neck	2 (2.9)	9 (4.3)	0.736
Gangrenous gallbladder	0	4 (1.9)	0.575
Gallbladder perforation	0	8 (3.9)	0.207
Sonographic murphy sign positive	2 (2.9)	24 (11.6)	0.057
Acute cholecystitis	6 (8.7)	49 (23.7)	0.012
Chronic cholecystitis	4 (5.8)	19 (9.2)	0.53
IHD of CBD dilation	8 (11.6)	37 (17.9)	0.301
Mirizzi syndrome/cholecystoenteric fistula	3 (4.3)	3 (1.4)	0.167
Pancreatitis	5 (7.2)	9 (4.3)	0.349
Liver cirrhosis	2 (2.9)	7 (3.4)	1
Cystic duct stone	11 (15.9)	51 (24.6)	0.183
Operative and postoperative outcome			
Complication^b^(Clavien-Dindo classification)	6 (8.7)	40 (19.3)	0.062
Grade I			
Surgical site infection	1 (1.4)	10 (4.8)	0.302
Wound dehiscence	0	2 (1)	1
Grade IIIa–b			
Postoperative CBD stone	1 (1.4)	6 (2.9)	0.684
Liver injury grade I	1 (1.4)	4 (1.9)	1
Bile duct injury^c^			
Major duct injury			
Hepatic duct injury	0	1 (0.5)	1
Minor duct injury			
Cystic duct injury	0	2 (1)	1
Cystic duct stump leakage	1 (1.4)	3 (1.4)	1
Postoperative bile leakage	0	1 (0.5)	1
Bowel injury	1 (1.4)	3 (1.4)	1
Postoperative intra-abdominal collection	1 (1.4)	6 (2.9)	0.684
Port-site hernia	1 (1.4)	2 (1)	1
Cystic artery injury	0	3 (1.4)	0.576
Hepatic vessel injury	0	1 (0.5)	1
Estimated blood loss, mL, median (range)	5 (5–10)	10 (5–30)	<0.001
Operative time, min, median(range)	115 (90–135)	145 (115–180)	<0.001
Conversion	0	6 (2.9)	0.342
30-day reoperation^c^	0	1 (0.5)	1
30-day readmission			
All-cause readmission	2 (2.9)	4 (1.9)	0.642
LC related	1 (1.4)	3 (1.4)	1

a Early and late hospital discharges were defined as discharge before and 24 h postoperatively, respectively.

b No complication in Clavien-Dindo classification grade II, V.

c A number of cases were not successfully matched during the propensity score matching process, leading to their exclusion from the subsequent analysis.

Data are presented as *n* (%) unless otherwise indicated.

ALP, alkaline phosphatase; BT, body temperature; CBD, common bile duct; ERCP, endoscopic retrograde cholangiopancreatography; IHD, intrahepatic duct; LC, laparoscopic cholecystectomy; SGOT, serum glutamate oxaloacetate transaminase; SGPT, serum glutamate pyruvate transaminase; WBC, white blood cell.

## Discussion

The reported incidence of major bile duct injuries ranges from 0.02 to 1.5%^20–23^. In this study, nine (0.4%) patients had major bile duct injuries, a rate consistent with other studies. Four patients underwent ERCP and stent insertion, three underwent suture repair, and two required hepaticojejunostomy reconstruction. Severe adhesions were a common operative finding in these cases. Minor bile duct injuries occurred in 22 (0.9%) patients, managed with either endoscopic stent insertion or surgical repair. These patients performed well during follow-up.

Significant vascular injuries, with a reported incidence of 0.03–0.06%, are the second most common cause of death after anaesthesia-related complications in patients undergoing LC^24^. However, none of the patients in this study had a significant vascular injury. The incidence of conversion to open surgery has been reported to range from 2.6 to 7.7%^1,25,26^; however, the conversion rate in this study was slightly lower at 2.4%, with severe adhesion as the primary reason. Reoperation within 30 days was necessary in four patients due to various complications: (i) bleeding from the gallbladder bed on day 0, (ii) hepatic flexure colon injury on day 1, (iii) ileal injury on day 2, and (iv) total transection of the common hepatic duct on day 10. One of the four patients developed cardiac arrest 6 h postoperation. The patient underwent reoperation after successful cardiopulmonary resuscitation and bleeding from the gallbladder bed was detected. Bleeding was stopped using multiple haemostatic techniques, including abdominal packing. However, the patient later died of multiorgan failure. This represents the only death (mortality rate 0.04%) in our study, compared to other studies where the mortality rates following LC ranged from 0.15% to 0.5%^27,28^. Twenty patients (0.87%) had LC-related readmissions within 30 days, which was quite low. A meta-analysis by McIntyre *et al.* ^29^ reported an overall readmission rate of 3.3% (range 0.0–11.7%). In our study, 55 patients (2.4%) developed surgical site infection (SSI) but were all successfully treated with antibiotics and wound care. This SSI rate aligns with the previously reported range of 0.6–3.2%^30–32^, emphasizing that SSI should be considered as a predisposing factor for TSH^33,34^. The incidence of TSH has been reported to be 0.2–1.7%^3,33,35^. Our study detected TSH in 13 patients (0.6%), a rate within the reported range. However, these hernias were located at the umbilical site, which differs from some studies reporting hernias at various sites, including the umbilical port, epigastrium, and right upper quadrant^3,33^.

A comparison of LC safety between low- and high-risk patients revealed a low incidence of LC-related complications in both groups. This study showed that the overall complication rate, operative time, conversion to open surgery, 30-day reoperation rate, and 30-day readmission rate were not significantly different between the groups. Although the median estimated blood loss was statistically significantly greater in the high-risk group, the difference was only 5 ml (10 vs. 5 ml), which is not clinically important. Comparative studies between different risk groups for non-ambulatory LC are relatively scarce, given the established safety of LC, as evidenced by a 30-year systematic review by Pucher *et al*. They reported a minimal morbidity range of 1.6–5.3%, mortality of 0.08–0.14%, and conversion rates between 4.2 and 6.2%^23^. Musbahi *et al.* found that the most common negative outcome in high-risk cases was readmission, occurring in 23 (9.43%) patients, while mortality was least common, occurring in two (0.82%) patients^5^.

When comparing the early and late discharge groups in the high-risk patient group, the percentage of overall complications in the late discharge group was more than twice that in the early discharge group (19.3% vs. 8.7%; *P*=0.062). This was most likely because a greater percentage of patients in the late discharge group had a history of acute cholecystitis (31.4% vs. 11.6%; *P*=0.002), acute cholecystitis based on imaging studies or intraoperative findings (23.7% vs. 8.7%; *P*=0.012), or multiple episodes of biliary colic (98.6% vs. 92.8%; *P*=0.025). Therefore, the operative time was significantly longer (145 vs. 115 min, *P*<0.001). Although complications from LC can impact the duration of hospital stay, operative time has been identified as a predictor or influential factor on postoperative hospital stay after LC^12,36,37^. Moreover, patients with a history of acute cholecystitis, cholangitis, or pancreatitis and patients with an abdominal drain or pain score greater than 3, severe postoperative pain, or postoperative nausea and vomiting had a longer hospital stay^37,38^. Despite differences in LC complications between the groups, there was no significant difference in complication rate, conversion to open surgery, 30-day reoperation, and 30-day readmission rates in this study. Furthermore, the slightly greater estimated blood loss (10 vs. 5 ml; *P*<0.001) in the high-risk group was not statistically significant. Previous studies align with our findings, reporting safety outcomes in ambulatory LC for both low-risk^11–13^ and high-risk patients^14,15^. This suggests that the duration of hospital stay can be reduced while maintaining safety standards in high-risk patients, thereby highlighting the potential for early discharge post-LC.

To achieve early discharge, implementation of a cholecystectomy surgery protocol designed for enhanced recovery, as suggested by a systematic review by Cole^39^ could be beneficial. Such a protocol could decrease the recovery time and hospital stay length for patients undergoing LC, thus improving day-case surgery success rates. Our results on LC safety outcomes in high-risk and low-risk patients provide compelling evidence to establish safe early discharge protocols in patients with LC to reduce hospital crowding, healthcare workload, overall costs, and patient stress^17^. Furthermore, a study on the financial benefits of an ambulatory program reported savings exceeding 300% per year^40^.

### Strengths and limitations

This study’s strengths include a large patient cohort from a single tertiary care hospital and the use of propensity score-matched analysis to balance variables and reduce bias. Limitations include the focus on surgical complications and the exclusion of other complications (e.g. pain, nausea/vomiting, abdominal drains) due to the retrospective design.

One patient, who had intended to undergo a LC, needed to terminate the procedure immediately upon entry into the abdomen due to vascular injury. However, since no LC-related operation had been initiated, this patient was also excluded from the study.

An important limitation of this study is that the analysis did not encompass the causes of late discharge among the cohort of 276 high-risk patients. As a result, the potential factors contributing to the late discharge were not thoroughly explored within the scope of this research. This limitation could influence a comprehensive understanding of the discharge process and its associated challenges in this specific population.

Missing data are the potential limitations as some patients sought care elsewhere before routine follow-up. Additionally, the sample size of the high-risk group may limit robust conclusions (1−β = 0.604). Future studies should include a larger number of high-risk patients for more definitive results.

## Conclusion

In conclusion, it is safe to perform LC in high-risk patients and discharge them early from the hospital. Nevertheless, a history of acute cholecystitis can prolong hospital stays due to longer operative times and increased estimated blood loss.

## Ethical approval

This study was approved by the Research Ethics Committee of the Faculty of Medicine, Prince of the Songkhla University. (REC 4607).

## Consent

Given the retrospective nature of our study, the need for informed consent was waived and approved by the Research Ethics Committee of the Faculty of Medicine, Prince of the Songkhla University. (REC 4607).

## Source of funding

None.

## Author contribution

P.B. contributed to the conceptualization, methodology, and data collection. K.S. performed the statistical analysis, data interpretation, and writing the manuscript. P.W. supervised the critical review and manuscript revision. K.Y. offered clinical expertise, data interpretation, and final approval of the manuscript. S.C. was the project administrator and provided overall guidance and final approval of the manuscript.

## Conflicts of interest disclosure

None.

## Research registration unique identifying number (UIN)

REC.65-455-10-1.

Declaration of Helsinki of The International Conference on Harmonization in Good Clinical Practice.

Research Registry.

https://www.researchregistry.com/registernow#home/registrationdetails/649e8ad3f3d9c70027e82d54/,

researchregistry9212.

## Guarantor

Siripong Cheewatanakornkul.

## Data availability statement

The datasets generated during and/or analyzed during the current study are publicly available.

## Provenance and peer review

Not commissioned or externally peer-reviewed.

## Supplementary Material
