# Supplementary material for: Propensity score-matched comparison of safety outcomes between high-risk and low-risk patients towards early hospital discharge after laparoscopic cholecystectomy
Source: Ann Med Surg (Lond). 2023 Sep 13;85(11):5337–43. doi: 10.1097/MS9.0000000000001300 (PMC10617936; doi:10.1097/MS9.0000000000001300)
Supplement: Supplementary file 1 [file ms9-85-5337-s001.docx]

**Supplement 1**

Classification of procedure-related postoperative complications according to the Clavien-Dindo classification.

| Clavien-Dindo classification* | **N = 2296** |
| --- | --- |
| Grade I |  |
| Surgical site infection | 55 (2.39) |
| Wound dehiscence | 4 (0.17) |
| Post cholecystectomy syndrome | 1 (0.04) |
| Abdominal wall infection | 1 (0.04) |
| Wound hematoma | 1 (0.04) |
| Grade II |  |
| Biliary pancreatitis | 6 (0.26) |
| Grade IIIa–b |  |
| Postop common bile duct stone | 41 (1.78) |
| Liver injury grade I | 35 (1.52) |
| Bile duct injury  Major duct injury  Common bile duct injury  Hepatic duct injury  Minor duct injury  Cystic duct injury  Cystic duct stump leakage  Duct of Luschka injury  Postop bile leakage | 31 (1.35)  5 (0.21)  4 (0.17)  9 (0.39)  8 (0.34)  3 (0.13)  2 (0.08) |
| Bowel injury | 17 (0.74) |
| Postop intraabdominal collection | 16 (0.69) |
| Port site hernia | 14 (0.61) |
| Cystic artery injury | 5 (0.21) |
| Hepatic vessel injury | 5 (0.21) |
| Postop cholangitis | 2 (0.08) |
| Right diaphragmatic injury | 1 (0.04) |
| Trocar site bleeding | 1 (0.04) |
| Umbilical injury | 1 (0.04) |
| Omental injury | 1 (0.04) |
| **Grade V** |  |
| Postoperative mortality | 1 (0.04) |
| Total | 244 (10.63%) |

Data are presented as n (%).

* No complications in Clavien-Dindo classification grade IV.
